# Arterite de Takayasu: A Idade é um Fator Diferencial no Diagnóstico, Acompanhamento e Tratamento da Doença?

**DOI:** 10.36660/abc.20220877

**Published:** 2023-01-31

**Authors:** Fernanda Luciano Rodrigues

**Affiliations:** 1 Universidade Federal de Mato Grosso do Sul Três Lagoas MS Brasil Universidade Federal de Mato Grosso do Sul, Campus de Três Lagoas, Três Lagoas, MS – Brasil

**Keywords:** Arterite de Takayasu, Glucocorticoides, Diagnóstico

A arterite de Takayasu (TAK) é uma vasculite inflamatória de grandes vasos, que afeta principalmente a aorta, seus principais ramos divisionais e as artérias pulmonares, e classicamente ocorre em mulheres de 20 a 40 anos.^1 - 3^ A TAK frequentemente se apresenta com sintomas inespecíficos como febre, fadiga, dor abdominal e perda de peso.^1 , 3^

A TAK é uma condição com a qual é difícil lidar. A identificação precoce desta doença é difícil e requer suspeita clínica e vigilância. Métodos radiológicos podem identificar vasos doentes, mas falham em distinguir entre lesões ativas e crônicas.^4^ A vasculite sistêmica pode levar a cicatrizes irreversíveis nos órgãos afetados, que podem ser causadas pela atividade da doença e/ou abordagens terapêuticas como corticosteroides e outros imunossupressores.^5^ Os glicocorticoides continuam sendo os mais eficazes e servem como tratamento de primeira linha fundamental.^6^

Como a TAK é uma condição típica de adultos jovens, há poucos estudos em populações fora dessa faixa etária. Poucos estudos sugerem que as fases do curso da TAK podem variar com a idade.^7 , 8^ Os autores do artigo “Particularidades dos Pacientes com Arterite de Takayasu em Idade Mais Avançada: Estudo Coorte, Retrospectivo e Transversal”^9^ relataram que, apesar do curso da TAK parecer variar com a idade, nenhum estudo até o momento avaliou comparativamente indivíduos adultos com TAK em idade mais avançada, levantando a questão das implicações e possíveis diferenças na abordagem dessa população.

O artigo^9^ é o primeiro relato de que pacientes idosos com TAK requerem tratamento medicamentoso mínimo para doença TAK subjacente e apresentam maior comprometimento do estado funcional. Considerando que estudos demonstraram que a população juvenil apresentou taxas de remissão mais baixas do que pacientes adultos,^8^ esses dados sugerem que a idade é um fator importante a ser considerado no diagnóstico, acompanhamento e tratamento de pacientes com TAK.

Segundo o artigo,^9^ menos pacientes idosos faziam uso de prednisona e imunossupressores ou imunobiológicos. Os glicocorticoides sistêmicos são o tratamento de primeira linha para TAK, geralmente iniciados em altas doses e seguidos por um regime de redução gradual.^10 , 11^ No entanto, a terapia com glicocorticoides é frequentemente associada a cicatrizes irreversíveis nos órgãos afetados,^5^ e seu tratamento crônico está associado a efeitos adversos graves, como diabetes, hipertensão, doença cardiovascular precoce, infecções e osteoporose.^12^

Os dados do artigo^9^ apontam que a população idosa pode não se beneficiar da terapia com glicocorticoides, o que por outro lado, seria benéfico, pois os idosos comumente apresentam comorbidades que podem ser agravadas pela terapia crônica com glicocorticoides.

Embora a população idosa requeira tratamento medicamentoso mínimo, o artigo^9^ demonstrou que pacientes mais velhos apresentam maior comprometimento do estado funcional. No entanto, o estudo não permite concluir se o impacto funcional foi causado pelo aumento da cronicidade da doença ou se está correlacionado com a idade avançada e a maior prevalência de comorbidades.

O estudo,^9^ pela primeira vez, destacou que pacientes idosos com TAK requerem diferentes abordagens, pois fatores relacionados à idade e à alta incidência de comorbidades podem levar à confusão no diagnóstico e avaliação da atividade da TAK. Além disso, o estudo reforça os achados da literatura de que as fases do curso do TAK variam com a idade e propõem que a idade seja um fator importante a ser considerado na abordagem dos pacientes TAK.
