# Supplementary material for: Arterite de Takayasu: A Idade é um Fator Diferencial no Diagnóstico, Acompanhamento e Tratamento da Doença?
Source: Arq Bras Cardiol. 2023 Jan 31;120(1):e20220877. [Article in Portuguese] doi: 10.36660/abc.20220877 (PMC10389112; doi:10.36660/abc.20220877)
Supplement: Translation

# Translation: Takayasu’s Arteritis: Is Age a Differential Factor in the Diagnosis, Follow-Up, and Treatment of the Disease?

**Authors:** Fernanda Luciano Rodrigues

**Affiliations:** 1 Brazil Universidade Federal de Mato Grosso do Sul, Campus de Três Lagoas, Três Lagoas, MS – Brazil

**Keywords:** Takayasu Arteritis, Glucocorticoids, Diagnosis

Takayasu’s arteritis (TAK) is a large-vessel inflammatory vasculitis, mainly affecting the aorta, its main divisional branches, and pulmonary arteries, being classically presenting in females aged 20 to 40 years.^1 - 3^TAK often presents with nonspecific symptoms such as fever, fatigue, abdominal pain, and weight-loss.^1 , 3^

TAK is a difficult condition to deal with. Early identification of this disease is difficult and necessitates clinical suspicion and vigilance. Radiologic methods can identify diseased vessels but fail to distinguish between active and chronic lesions.^4^Systemic vasculitis can lead to irreversible scars in affected organs, which may be caused by disease activity and/or therapeutic approaches such as corticosteroids and other immunosuppressives.^5^Glucocorticoids remain the most effective and serve as a cornerstone first-line treatment.^6^

Since TAK is a typical condition of young adults, there are few studies in populations outside this age range. Few studies suggest that the life stages of the TAK course can vary with age.^7 , 8^The authors of the article “Characteristics of Older Patients with Takayasu’s Arteritis: A Two-Center, Cross-Sectional, Retrospective Cohort Study”^9^related that despite the TAK’s course seeming to vary with age, no studies to date have comparatively evaluated adult individuals with TAK at an older age, raising the question of the implications and possible differences in the approach to this population.

The article^9^is the first report that older patients with TAK require minimal drug treatment for underlying TAK disease and have greater impairment of functional status. Considering that studies have demonstrated that the juvenile population had lower remission rates than adult patients,^8^these data suggest that age is an important factor to consider in the diagnosis, follow-up, and treatment of TAK patients.

According to the article,^9^fewer older patients used prednisone and immunosuppressive or immunobiological drugs. Systemic glucocorticoids are the first-line treatment for TAK, usually started at high doses and followed by a tapering regimen.^10 , 11^However, glucocorticoid therapy is frequently associated with irreversible scars in affected organs,^5^and their chronic treatment is associated with severe adverse effects, such as diabetes, hypertension, early cardiovascular disease, infections, and osteoporosis.^12^

The article^9^data point out that the elderly population may not benefit from glucocorticoid therapy, which would be beneficial since the elderly commonly have comorbidities that can be aggravated by chronic therapy with glucocorticoids.

Even though the elderly population requires minimal drug treatment, the article^9^demonstrated that older patients have greater impairment of functional status. However, the study does not allow us to conclude if the functional impact was caused by an increase in the chronicity of the disease or is correlated with advanced age and a higher prevalence of comorbidities.

The study,^9 ,^for the first time, highlighted that elderly TAK patients require different approaches since age-related factors and the high incidence of comorbidities can lead to confusion in diagnosing and assessing TAK’s activity. In addition, the study reinforces the findings in the literature that the life stages of the TAK course vary with age and propose that age is an important factor to be considered in the approach to TAK patients.
